# Epidemiological and entomological studies of a malaria outbreak among French armed forces deployed at illegal gold mining sites reveal new aspects of the disease’s transmission in French Guiana

**DOI:** 10.1186/s12936-016-1088-x

**Published:** 2016-01-22

**Authors:** Vincent Pommier de Santi, Romain Girod, Marie Mura, Aissata Dia, Sébastien Briolant, Félix Djossou, Isabelle Dusfour, Alexandre Mendibil, Fabrice Simon, Xavier Deparis, Frédéric Pagès

**Affiliations:** French Armed Forces Center for Epidemiology and Public Health (CESPA), Camp Militaire de Sainte Marthe, BP 40026, 13568 Marseille Cedex 02, France; Direction Interarmées du Service de Santé en Guyane, Quartier La Madeleine, BP 6019, 97306 Cayenne Cedex, French Guiana; Medical Entomology Unit, Institut Pasteur de la Guyane, 23 Avenue Pasteur, BP 6010, 97306 Cayenne Cedex, French Guiana; Institut de Recherche Biomédicale des Armées, BP 73, 91223 Brétigny sur Orge Cedex, France; Laboratory of Parasitology, Institut Pasteur de la Guyane, 23 Avenue Pasteur, BP 6010, 97306 Cayenne Cedex, French Guiana; Unit of Infectious and Tropical Diseases, Andrée Rosemon Hospital, Avenue des Flamboyants, Cayenne, French Guiana; Antenne médicale de Castres, Quartier Fayolle – 68 avenue J. Desplat, CS 50025, 81108 Castres Cedex, France; Department of Infectious Diseases and Tropical Medicine, Laveran Military Teaching Hospital, 34 Boulevard Laveran, BP 50, 13013 Marseille, France; Cire Océan Indien, Institut de Veille Sanitaire, 2 bis, av Georges Brassens, CS 61002, 97743 Saint-Denis Cedex 9, Réunion, France

**Keywords:** Malaria, French Guiana, Illegal gold mining, Military, *Plasmodium vivax*, Outbreak, *Anopheles darlingi*, *Anopheles marajoara*

## Abstract

**Background:**

In December 2010, a *Plasmodium vivax* malaria outbreak occurred among French forces involved in a mission to control illegal gold mining in French Guiana. The findings of epidemiological and entomological investigations conducted after this outbreak are presented here.

**Methods:**

Data related to malaria cases reported to the French armed forces epidemiological surveillance system were collected during the epidemic period from December 2010 to April 2011. A retrospective cohort study was conducted to identify presumed contamination sites. *Anopheles* mosquitoes were sampled at the identified sites using Mosquito Magnet and CDC light traps. Specimens were identified morphologically and confirmed using molecular methods (sequencing of ITS2 gene and/or barcoding). *Anopheles* infections with *Plasmodium falciparum* and *P. vivax* were tested by both enzyme-linked immunosorbent assay and real-time PCR.

**Results:**

Seventy-two *P. vivax* malaria cases were reported (three were mixed *P. falciparum*/*P. vivax* infections), leading to a global attack rate of 26.5 % (72/272). Lack of compliance with vector control measures and doxycycline chemoprophylaxis was reported by patients. Two illegal gold mining sites located in remote areas in the primary forest were identified as places of contamination. In all, 595 *Anopheles* females were caught and 528 specimens were formally identified: 305 *Anopheles darlingi*, 145 *Anopheles nuneztovari* s.l., 63 *Anopheles marajoara* and 15 *Anopheles triannulatus* s.l. Three *An. darlingi* were infected by *P. falciparum* (infection rate: 1.1 %) and four *An. marajoara* by *P. vivax* (infection rate: 6.4 %).

**Discussion:**

The main drivers of the outbreak were the lack of adherence by military personnel to malaria prevention measures and the high level of malaria transmission at illegal gold mining sites. *Anopheles marajoara* was clearly implicated in malaria transmission for the first time in French Guiana. The high infection rates observed confirm that illegal gold mining sites must be considered as high level malaria transmission areas in the territory.

**Conclusions:**

Illegal gold mining activities are challenging the control of malaria in French Guiana. Collaboration with neighbouring countries is necessary to take into account mobile populations such as gold miners. Malaria control strategies in the French armed forces must be adapted to *P. vivax* malaria and sylvatic *Anopheles* species.

## Background

**Fig. 1:**
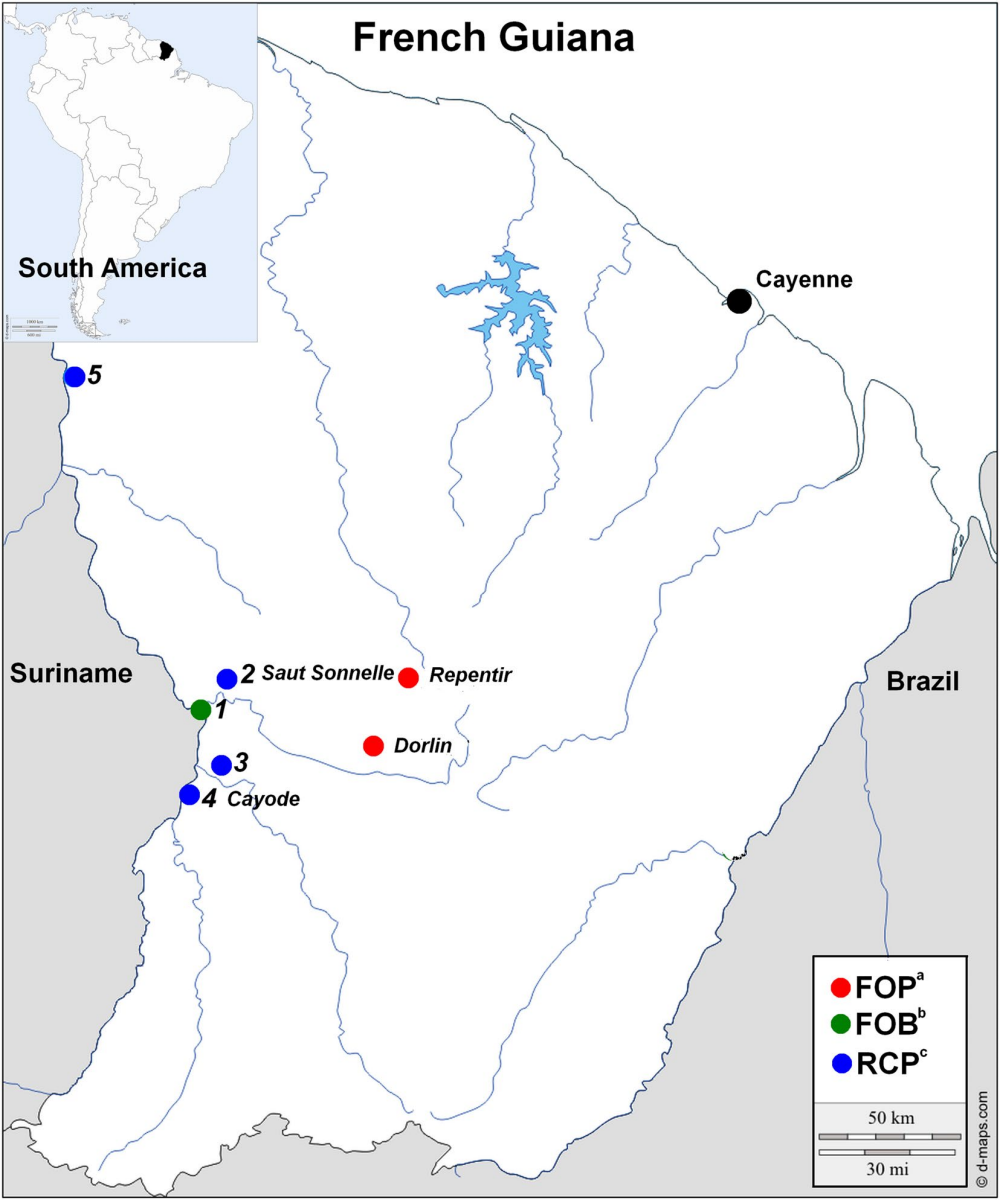
Map of French Guiana and location of French military units in 2010–2011. Legend: ^a^ *FOP* forward operational post, ^b^ *FOB* forward operational base, ^c^ *RCP* river check point. *1*—Maripasoula FOB, *2*—Saut Sonnelle RCP, *3*—Cayodé RCP, *4*—Twenké RCP, *5*—Providence RCP. Names in *italics* correspond to mosquito sampling sites

French Guiana is a French overseas entity and an Outermost Region of the European Union located on the northeast coast of South America (Fig. 1). It is a sparsely populated area—250,000 inhabitants in 2013—and 85 % of the territory is covered by the Amazon rainforest [1]. An estimated 80 % of the population lives on or close to the coastal area, characterized by malaria cases imported from the interior and, more rarely, indigenous transmission [2]. From 2005 to 2014, the number of malaria cases officially reported in French Guiana (all *Plasmodium* species) decreased from 4479 to 445 [3]. Most of these officially-reported cases currently occur in villages located along the main rivers flowing through the territory, especially those bordering Suriname and Brazil [3]. This is partially offset by an increase in the number of cases related to transmission at illegal gold mining sites. Indeed, such sites located in forested inland French Guiana are probably areas of malaria transmission for *Plasmodium vivax*, *Plasmodium falciparum* and *Plasmodium malariae* [4–7].

In 2009, Suriname estimated that 1140 cases of malaria diagnosed and treated in its clinics were imported cases, mainly from illegal gold mining sites in French Guiana [8]. In 2014, for the border town of Oiapoque (Oyapock River, Brazil), 21.1 % of malaria cases reported were imported from French Guiana, mostly related to illegal gold mining [9]. In the Amazon, the transmission of malaria due to human migration has been called “frontier malaria” [10]. The impact of illegal gold mining on malaria transmission has been highlighted in most parts of the Amazon Basin [11–14].

In French Guiana, *Anopheles* (*Nyssorhynchus*) *darlingi*, known for its anthropophilia and its adaptation to anthropized environments, is considered as the main vector of malaria [15–18]. In the Amazon Basin, it is described as biting all night with two peaks of activity at dusk and dawn. It can enter shelters or homes to take a blood meal, but rests afterwards in the forest [19–22]. Its breeding sites are linked to river banks and flooded areas when rivers overflow during the rainy season [23, 24]. In addition to *An. darlingi*, other anopheline species of the subgenera *Nyssorhynchus* and *Anopheles* have been incriminated in malaria transmission in the Amazon Basin [24–26]. Moreover, *Anopheles* of the subgenus *Kerteszia* are present in French Guiana. These species have been described as biting during the day along forest trails, and they have been suspected of playing a role in malaria transmission [27].

Because of illegal activity and violence, no entomological study has ever been conducted to date at illegal gold mining sites in French Guiana. French military personnel have been involved in a specific military operation to control and reduce illegal gold mining activities in French Guiana since 2005. The main purpose of this operation is to disrupt the logistics of illegal gold mining in the primary forest. To that end, 400 military personnel are permanently deployed at river checkpoints (RCP), forward operational bases on major rivers (FOB) and forward operational posts deep in the forest (FOP) (Fig. 1). Most of them come directly from France for 4-month missions in French Guiana. From forward bases, military personnel conduct short (24–48 h, camping in the forest) or long-term (3–4 weeks) interventions at illegal gold mining positions. During these missions, the malaria prevention strategy in the French armed forces is based on three axes: (1) Health education to encourage compliance with protective measures, (2) Personal protection against vectors including repellents (DEET or picaridine), use of permethrin-impregnated combat uniforms, long trousers and long-sleeved shirts from dusk to dawn (patrols and guards), deltamethrin-impregnated cotton bed nets in forward bases and hammocks with mosquito nets when camping deep in the forest (individual vector-bite protection equipment is provided to each soldier) and (3) Chemoprophylaxis based on 100 mg of doxycycline daily during their entire stay and for 4 weeks after leaving the malaria transmission area (terminal prophylaxis) [28]. Despite such strategies, deployments at illegal gold mining sites have resulted in several outbreaks and in an increase of malaria incidence among French forces, with *P. vivax* accounting for more than 80 % of reported cases [29].

In December 2010, the French military health surveillance system detected a *P. vivax* outbreak among three military units deployed from France to French Guiana for a 4-month mission. To explain this outbreak, a case series study was first conducted to describe the outbreak, followed by a retrospective cohort study to determine areas at risk for malaria transmission. In a second stage, an entomological study was conducted in the determined areas to identify the vectors involved and determine the level of malaria transmission.

## Methods

### Epidemiological investigation

Data were collected during the epidemic period from December 2010 to April 2011. A malaria case was mandatorily defined as any pathologic event or symptom associated with confirmed parasitological evidence (*Plasmodium* spp. in blood smears or quantitative buffy coat or positive malaria rapid diagnosis tests) contracted in French Guiana. Cases were reported to the French armed forces epidemiological surveillance system in French Guiana (indigenous cases) and in continental France for military units that had returned to France (imported cases). During missions, the French armed forces malaria prevention strategy was implemented [28]. The hammocks used were made of a single thin layer of fabric with a non-treated polyester mosquito net. For each malaria case, military physicians had to complete a mandatory specific form containing administrative, geographic and clinical information, compliance with individual protection measures against mosquito bites (repellent applied to skin, use of permethrin-impregnated combat uniform and bed or hammock net), compliance with chemoprophylaxis and biological data [29]. The level of compliance with vector control measures was assessed as either “never”, “seldom”, “often” or “always”, and proper compliance was defined as “always”. Chemoprophylaxis compliance was assessed as a daily 100 mg dose of doxycycline during the 8 days preceding a malaria attack. A retrospective cohort study was conducted to identify places of stay related to a *P. vivax* attack during the 4-month mission. These different locations were available for the two main military units affected by the epidemic.

### Entomological investigation

#### Mosquito field sampling

Entomological investigation in the field was guided by epidemiological investigation results. *Anopheles* mosquitoes were sampled from June to July 2011. As classic human landing collections could not be conducted, different traps were used: Mosquito Magnet^®^ (MM) traps (Woodstream Corporation, Lititz, PA) and Center for Disease Control and Prevention (CDC) light traps. One MM trap baited with octenol was deployed along the Maroni River (border with Suriname) at the two river checkpoints of Cayodé and Saut Sonnelle, for 1 month (Fig. 1). Traps were checked after dawn and before dusk every day. Two mosquito collection sessions were conducted at two illegal gold mining sites: Repentir from June 9 to 15, and Dorlin from June 24 to July 15 (Fig. 1). Due to safety and logistical reasons, French military personnel accompanied the research team in the field. Each time, a medical entomologist was present to install traps during the first nights. Three MM traps baited with octenol were in use night and day during the stay and checked after dawn and before dusk. Two CDC light traps were used, but only for the first three nights due to the short lifespan of batteries in the rainforest and logistical limitations.

All mosquitoes were stored individually in numbered vials with desiccant, and were preserved at −20 °C until processing at the medical entomology unit of the Institut Pasteur de la Guyane (IPG), Cayenne (French Guiana), or at the medical entomology unit of the Institute for Biomedical Research of the French Armed Forces (IRBA), Marseille (France). Before storage at −20 °C, mosquitoes were conserved at ambient temperature in the rainforest for 1–15 days according to the date of collection.

Water collections in the study areas were examined for anopheline larvae. Larval surveys were conducted in the immediate vicinity of the settlements. Larvae and pupae were sampled using a standard dipping method in water collections [30]. Bromeliads recovered from fallen trees were also explored for larvae of the subgenus *Kerteszia*. Larvae and pupae were reared in the field. The neonate adult mosquitoes were stored by date and breeding site in numbered vials with desiccant and preserved at −20 °C, until processing.

#### *Anopheles* identification

Adult mosquitoes collected or neonate mosquitoes were sorted by genera, and *Anopheles* specimens were morphologically identified based on keys in use in the Guiana Shield [31–34].

Mosquitoes caught with traps are often in bad condition, and some specimens are difficult to identify [35]. Furthermore, as no catches had ever been conducted at some of the selected sampling sites, species never described in French Guiana but present in neighbouring countries may have been present deep in the forest. Morphological identifications were then completed by molecular analysis following established protocols. Mosquito DNA was extracted, Internal transcribed spacer 2 (ITS2) gene was processed by PCR-RFLP and sequenced for a selected sample and for specimens infected by *P. falciparum* or *P. vivax* [36, 37]. DNA barcodes were also used for the final identification of some specimens [38].

#### *Plasmodium falciparum* and *Plasmodium vivax* detection

Heads and thoraces of a sample of *Anopheles* females were tested by enzyme-linked immunosorbent assay (ELISA) for *P. falciparum* and *P. vivax* strains VK210 and VK247 circumsporozoite protein (CSP) [39]. However, using ELISA to research human *Plasmodium* infection of the *Anopheles* captured in deep forest has two major drawbacks: the lack of a cold chain and the possibility of non-specific cross-reactions with non-human *Plasmodium*. The lack of a cold chain leads to a deterioration of parasite proteins detected by ELISA, which could explain the lack of detection of infected *Anopheles* mosquitoes. Furthermore, zoophilic *Anopheles* can be infected by “animal” *Plasmodium* that could generate false positives by cross reaction and thus be considered of importance to human health [40]. Therefore, a RT-PCR detection of *Plasmodium* infection was systematically carried out for each *Anopheles* specimen [41]. The CSP index for each *Plasmodium* species was calculated as the proportion of mosquitoes found to be positive for CSP or for *Plasmodium* DNA.

### Statistical analysis

Univariate analysis was conducted to determine places of stay during the 4-month mission related to the risk of malaria. Relative risk of contracting malaria was calculated for each place of stay. The results are expressed as a percentage of subjects for incidence and attack rates and as a percentage for compliance with protection measures. Excel and SAS 9.3^®^ software were used. Geographic maps used were free open access [42].

## Results

### Epidemiological investigation

**Fig. 2:**
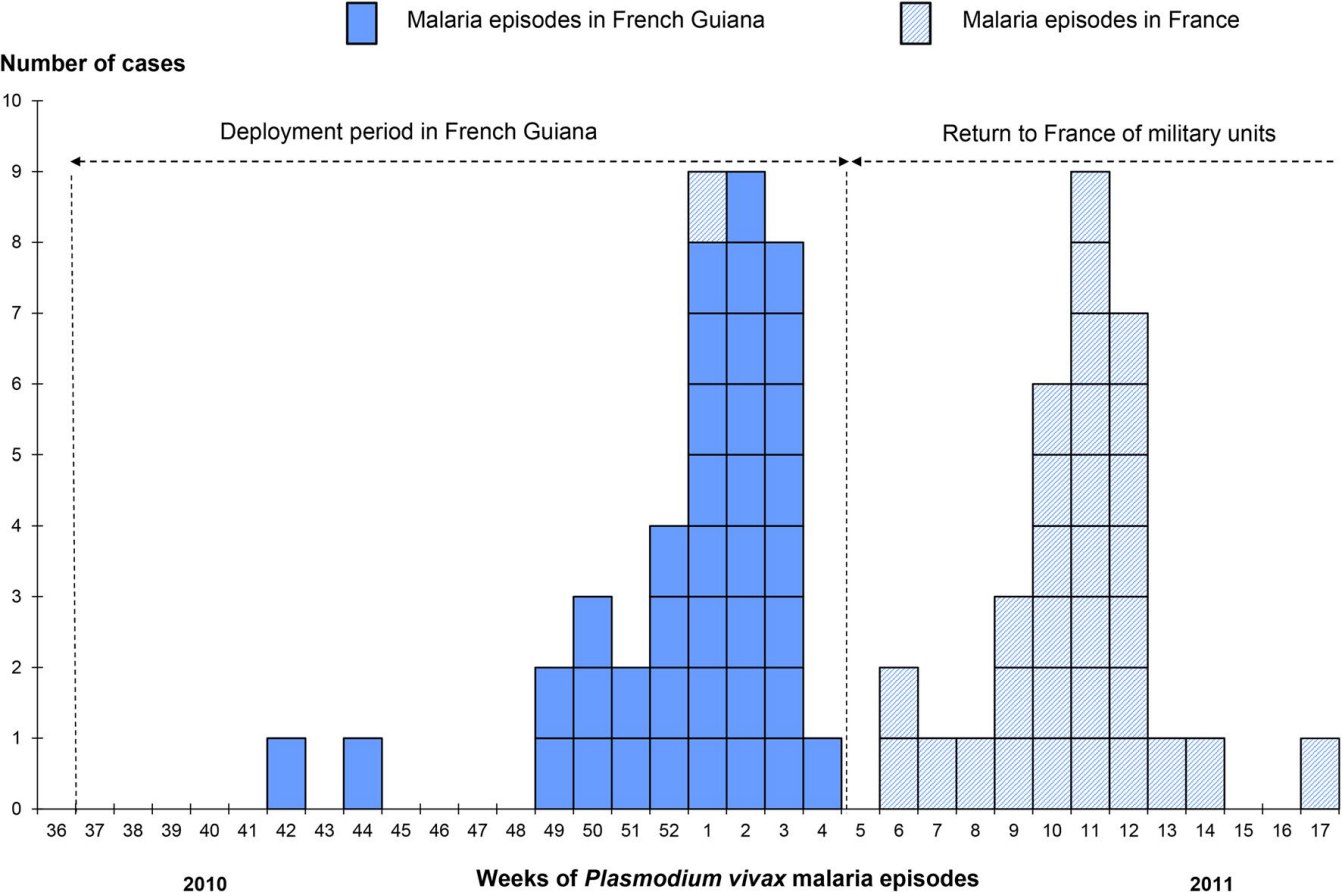
Epidemic curve of *Plasmodium vivax* episodes among French military personnel 2010–2011 (N = 72)

The epidemic lasted 7 months, with a total of 72 *P. vivax* malaria cases, including three mixed *P. falciparum*/*P. vivax* infections. Three military units (N = 272) deployed for 4 months (September 2010–January 2011) were affected by the epidemic: one infantry company (n = 138), one sapper company (n = 116) and one platoon of Gendarmes (n = 18) (Fig. 2). These three units had joint missions during their stay in French Guiana. The first *P. vivax* malaria case occurred on October 21, 2010, 7 weeks after deployment to French Guiana, and the last case on April 28, 2011, 4 months after returning to France (Fig. 2). The overall attack rate was 26.5 % (72/272). Attack rates for the infantry company, the sapper company and the platoon of Gendarmes were comparable: 24.6 % (34/138), 28.4 % (33/116), 27.8 % (5/18), respectively. The epidemic curve had two peaks, the first at the end of the mission in French Guiana (indigenous cases) and the second after the soldiers’ return to France (imported cases) (Fig. 2). Indigenous cases accounted for 54.2 % (39/72) and imported cases for 45.8 % (33/72). Malaria occurred with a median delay of 36 days [interquartile interval (28–86 days)] after returning to France. In all, 82 % (27/33) of imported cases occurred after terminal doxycycline chemoprophylaxis. One of the patients presented a malarial splenomegaly and a splenic rupture after a seemingly harmless abdominal trauma.

Compliance with personal protection measures against mosquito bites was completed for 61 cases. Bed or hammock nets were always used by 59 % (36/61), insect repellents by 46 % (28/61) and permethrin-impregnated combat uniforms by 52 % (32/61) of the cases. Several of the patients stated that they had been bitten through the cloth of their hammock during nights spent in deep forest. Evaluation of chemoprophylaxis compliance was not relevant for malaria attacks occurring more than 28 days after returning to France (n = 27). But for other cases, proper compliance (patient’s self-evaluation) with doxycycline in the 8 days preceding a malaria attack was 40 % (18/45).

Places of stay during the 4-month mission in French Guiana were available for infantry and sapper companies (N = 254). Two locations were associated with malaria: the illegal gold mining sites of Dorlin [relative risk = 2.68 CI 95 % (1.61–4.46)] and Repentir [relative risk = 5.35 CI 95 % (3.08–9.30)] (Table 1; Fig. 1).

**Table 1:** Places of stay in French Guiana related to *Plasmodium vivax* attack among French military forces (N = 254, univariate analysis)

	N	Nb *P. vivax*	Incidence (%)	RR	CI 95 %	*p* value
Dorlin						*0.0117*
No	245	61	24.9	1		
Yes	9	6	66.7	*2.68*	*1.61–4.46*	
Repentir						*<0.0001*
No	143	13	9.1	1		
Yes	111	54	48.6	*5.35*	*3.08–9.30*	
Twenké						0.2760
No	213	59	27.7	1		
Yes	41	8	19.5	0.70	0.36–1.36	
Cayodé						0.2631
No	196	55	28.1	1		
Yes	58	12	20.7	0.73	0.42–1.28	
Maripasoula						0.9236
No	35	9	25.7	1		
Yes	219	58	26.5	1.03	0.56–1.89	
Saut Sonnelle						0.4119
No	136	33	24.3	1		
Yes	118	34	28.8	1.19	0.79–1.79	
Providence						0.3565
No	219	60	27.4	1		
Yes	35	7	20.0	0.73	0.36–1.47	

Statistically significant results are in italics

### Entomological investigation

#### Mosquito collection and identification

During the survey in June and July, 595 female *Anopheles* were caught only at night, including 554 specimens at the two illegal gold mining sites and 41 specimens at both river checkpoints (40 with MM at Cayodé, one with CDC light trap at Saut Sonnelle). In Repentir, 166 *Anopheles* mosquitoes were caught: 164 using two MM traps over the entire period and two using two CDC traps for three nights. In Dorlin, 388 *Anopheles* mosquitoes were caught: 370 using two MM traps over the entire period and 18 using two CDC traps for three nights. Morphological identification was difficult due to the damage caused to specimens by traps and the presence of moisture. It allowed the probable species identification of 398 specimens: 249 *Anopheles darlingi*, 88 *Anopheles nuneztovari* s.l., 53 *Anopheles albitarsis* s.l. and seven *Anopheles triannulatus* s.l. Morphological identification was inconclusive for 199 specimens (199 *An.* spp.) (Table 2). Molecular identification by ITS2 PCR-RFLP made it possible to formally identify 528 specimens: 305 *An. darlingi*, 145 *An. nuneztovari* s.l., 63 *An. albitarsis* s.l. and 15 *An. triannulatus* s.l. (Table 2). For 27 specimens (six *An. triannulatus* s.l., seven *An. albitarsis* s.l., nine *An. triannulatus* s.l. and five *An. darlingi*), the products of ITS2 PCR were sequenced and compared to available sequences. The sequencing confirmed the molecular identification for 26 specimens. The overall consistency of morphological identification was 91 %. Table 3 summarizes the distribution of the species processed with ITS2 PCR-RFLP by location. Furthermore, six members of the *An. albitarsis* complex caught were molecularly identified using DNA barcoding. At the two sites where they were caught, all specimens were *Anopheles marajoara* [43].

**Table 2:** Distribution by species of the 595 anopheles caught in French Guiana in June–July 2011

Molecular identification	Morphological identification					
	*An. albitarsis* s.l.	*An. darlingi*	*An. nuneztovari* s.l.	*An. triannulatus* s.l.	*An.* spp.	Total
*An. albitarsis* s.l.	*48 (90.5* *%)*	4			11	63
*An. darlingi*	3	*224 (90.0* *%)*	1		77	305
*An. nuneztovari* s.l.		2	*83 (94.3* *%)*	1	59	145
*An. triannulatus* s.l.	1		3	*6 (85.7* *%)*	5	15
*An.* spp.		19	1		47	67
Total of KI	52	249	88	7	199	595

In italic values between parentheses, consistency between morphological identification and molecular analysis for each species

**Table 3:** Distribution by site of sampling of the 595 anopheles caught in French Guiana from June to July 2011

	*An. marajoara*	*An. darlingi*	*An. nuneztovari* s.l.	*An. triannulatus* s.l.	*An.* spp.	Total
Dorlin	62	262	20	2	42	388
Repentir		11	125	13	17	166
Cayodé		32			8	40
Saut Sonnelle	1					1
Total	63	305	145	15	67	595

#### *Plasmodium falciparum* and *Plasmodiumvivax* detection

Homogenates of the heads and thoraces of a sample of 345 *Anopheles* females were tested by enzyme-linked immunosorbent assay (ELISA) for *P. falciparum andP. vivax* strainVK210 and VK247 circumsporozoite protein. According to ELISA, none of those specimens was infected by a human *Plasmodium*. All the specimens were tested by RT-PCR for *P. falciparum* and *P. vivax*. DNA was extracted directly for 243 specimens not tested using ELISA and using the homogenates prepared for ELISA for the 345 others. By RT-PCR, infected anophelines were only found among samples from Dorlin. Three *An. darlingi* were infected by *P. falciparum* (infection rate of 1.1 %) and four *An. marajoara* were infected by *P. vivax* (infection rate of 6.4 %) (Table 4).

**Table 4:** *Anopheles* species collected in Dorlin and Circumsporozoite Protein Index for *P. vivax* and *P. falciparum*

Anopheline species	MM traps^a^	CDC traps^b^	*P. falciparum*		*P. vivax*	
			n	CSP^c^ (%)	n	CSP^c^ (%)
*An. darlingi*	262		3	1.1	0	–
*An. marajoara*	44	18	0	–	4	6.4
*An. nuneztovari* s.l.	20		0	–	0	–
*An. triannulatus* s.l.	2		0	–	0	–
*An.* spp.	42		0	–	0	–
Total	370	18	3	1.1	4	6.4

^a^Mosquito Magnet^®^ traps baited with octenol

^b^Centers for Disease Control and Prevention light traps

^c^Circumsporozoite Protein index

### Larvae collection

In Repentir, larval prospecting was done in ponds, pools, puddles and all-terrain vehicle (ATV) ruts. Some bromeliads were recovered from fallen trees. *Anopheles* larvae were found only in two ATV ruts filled by rainfall. They were reared in the field, but only one adult emerged, morphologically identified as *An. nuneztovari* s.l., with identification confirmed by ITS2 PCR-RFLP. No *Anopheles* larvae were found in the different bromeliads. In Dorlin, larval prospecting was done in ponds, pools, puddles and ATV ruts around the illegal settlement. No larvae were found.

## Discussion

This study reports on one of the largest malaria outbreaks among French forces deployed to French Guiana [44, 45]. For the first time, an entomological study was conducted in the field, particularly at illegal gold mining sites, to evaluate infection rates and characterize *Anopheles* species implicated in malaria transmission.

### Epidemiological investigation

High *P. vivax* malaria attack rates, ranging from 24.6 to 27.8 %, were observed in the three military units conducting the same mission: to control illegal gold mining in French Guiana. The epidemic curve presents two peaks.

The first peak corresponds to indigenous malaria cases and could be partly attributed to a lack of adherence to doxycycline chemoprophylaxis. Only 40 % of the patients stated proper chemoprophylaxis compliance in the 8 days before onset of symptoms. Comparable results had already been observed in previous malaria outbreaks among French forces [46, 47]. In a large prospective study conducted on 19 French military units, assessment of levels of compliance with chemoprophylaxis was based on the self-reported daily medication doses missed during the mission, and the average prevalence of appropriate compliance was estimated at 46.2 % [46]. Direct observation therapy is not used in the French forces [28]. During missions in the forest, taking doxycycline is more closely supervised by commanders in direct contact with soldiers. Most of the indigenous cases occurred at the end of the 4-month mission, when soldiers returned from the forest to the coast, which is free of malaria and where taking daily doses of doxycycline becomes an individual behaviour. The perception of lower malaria risk and the lack of group support at the end of the mission could explain poor levels of compliance with chemoprophylaxis [46]. Doxycycline has been shown to be highly effective as a blood schizonticidal agent, killing erythrocytic stages of the malaria parasite [48, 49]. But due to doxycycline’s short half-life and the mechanism of action, its efficiency depends on proper compliance.

The second peak occurred after leaving French Guiana, with 82 % of *P. vivax* malaria imported cases occurring after terminal doxycycline chemoprophylaxis. These patients did not experience *P. vivax* malaria or fever during the 4-month mission, and their first clinical episodes were probably hypnozoite-induced relapses. Doxycycline does not affect the liver stage of the parasite, which also means that it does not kill *P. vivax* hypnozoites and, therefore, does not prevent *P. vivax* malaria relapses [48, 50, 51]. Considering *P. falciparum* resistance to malaria drugs, and in order to optimize compliance with a homogeneous prescription, 100 mg of doxycycline once a day is currently the first line chemoprophylaxis in the French armed forces [28]. Malaria chemoprophylaxis strategies in the French armed forces are mainly aimed at preventing *P. falciparum* malaria during deployments to Africa. The involvement of French forces in endemic *P. vivax* malaria transmission areas is more recent and has led to an increase in *P. vivax* incidence [29]. The Australian Defence Force encountered the same problem with *P. vivax* relapses when they became involved in the United Nations mission in East Timor from 1999 to 2000 [52, 53]. Considering the lack of prophylaxis activity of doxycycline against *P. vivax*, a daily dose of 100 mg of doxycycline during operations and 22.5 mg of primaquine daily for 2 weeks on returning to Australia were administered [54]. Attack rates observed in their forward battalions ranged from 5 to 13 %, well below those observed in the present study (>25 %). Similarly, in 2006, CDC experts recommended presumptive anti-relapse therapy (terminal prophylaxis) with primaquine for 2 weeks in persons heavily exposed to *P. vivax* [55]. However, the French National Agency for Medicines and Health (ANSM) has limited the use of primaquine to patients with *P. vivax* attacks in order to prevent relapses. Therefore, liver-stage prophylaxis with primaquine for delayed-onset malaria cannot currently be used by French forces.

Scrupulously observing individual protection measures against mosquito bites is essential in such contexts. Their combined proper use will theoretically reduce exposure to biting mosquitoes, but not enough to significantly reduce the incidence of malaria among non-immune troops [56, 57]. Military personnel deployed in the interior of French Guiana live in wooden houses with corrugated aluminium roofs, without ceilings or windows. During missions in the forest, they stay directly at illegal gold mining sites, sleeping in hammocks under a waterproof tarp. Poor compliance with personal protection measures against mosquitoes was observed during missions. It suggests a failure by commanders in direct contact with soldiers to apply French armed forces health recommendations. However, hammock protection failure (mosquito bites through the fabric of the hammock during the night) most certainly played an important role in this epidemic.

### Entomological investigation

The main entomological investigations were conducted at Repentir and Dorlin. Both sites were identified as presumed contamination locations by the epidemiological investigation. These sites have been subjected to human pressure for many years. After an initial period of legal activity, the mining site of Repentir was rehabilitated before being closed. But the area was then invaded by undocumented gold miners. In December 2010, military intelligence estimated that more than one thousand undocumented people were living and working in this area. The river was diverted for gold mining purposes and ponds and puddles were created. The forest was cleared for gold mining and many excavations were made that were then flooded by the river or filled by rainfall. In some places, the primary forest was destroyed and replaced by savannas, secondary forest or glades. Wells were also dug. The rest of the surrounding primary forest was criss-crossed by footpaths and ATV tracks. In late 2010, French military forces took up positions in this area and undocumented workers left to the nearest illegal gold mining site: Dorlin. Dorlin is an old illegal gold mining site that has been exploited for 20 years. No rehabilitation of the area has ever been carried out. Military intelligence estimated that more than one thousand undocumented people were present here and working at this illegal gold mining site. Many illegal settlements were still present on the site during entomological investigations. Rivers have been diverted; the primary forest has been extensively cleared, replaced by savanna or secondary forest over large areas. Mining excavations have been transformed into lakes and ponds. Backhoes and bulldozers have been used to dig hills in the search for auriferous veins and footpaths and ATV tracks criss-cross the area.

Current entomological investigations confirm that these anthropic modifications are highly favourable to the development of *Anopheles* mosquitoes. Adult *Anopheles* from four species that could play a role in malaria transmission were collected with traps, in high densities in some places and periods. Larval prospecting was not significant due to the large size of the areas, the high number of potential breeding sites and the limitations of entomological activities due to security measures.

Morphological and molecular analysis made it possible to identify 89 % (528/595) of the *Anopheles* mosquitoes caught. Species identified were among the most frequently observed *Anopheles* species in French Guiana and the Amazon Basin [31]. However, this study confirms for the first time the role of *An. marajoara* as a *P. vivax* vector in the French Guiana rainforest. Infected anophelines were found only at the illegal gold mining site of Dorlin. The high infection rates observed confirm that illegal gold mining sites in French Guiana should be considered as high level malaria transmission areas. Thanks to the setting up of a forward operational post and legal mining operations, Repentir site was free of undocumented gold miners for 6 months before the entomological investigation. This could explain the absence of infected anopheles in this area. However, during the 1-week mission spent in Repentir, one *P. falciparum* malaria case occurred in a legal gold mining operation close to the FOP, suggesting a continuation of low malaria transmission. The use of traps did not enable the calculation of entomological inoculation rates (EIRs) or make it possible to clearly measure the number of infected bites per soldier in Repentir or Dorlin. Nevertheless, the number of *Anopheles* mosquitoes caught with traps at those sites was very high compared to previous studies in French Guiana. This suggests that anopheles densities were very high at these sites. Furthermore, CDC light traps that seemed to be more attractive to *An. marajoara* were only used for 3 days, and the abundance of this species was probably underestimated. Considering this, the malaria transmission risk could be considered as very high both at Dorlin and Repentir. These findings are consistent with other studies conducted at illegal gold mining sites including a study in Venezuela where the *An. marajoara* and *An. darlingi* association has already been described [58]. The combination of poor adherence of soldiers to protective measures and a stay in an area with a high level of malaria transmission explained the outbreak.

Illegal gold mining has modified the epidemiology of malaria in French Guiana, which was previously limited to the villages located on the main rivers flowing into the territory, in particular the Maroni and Oyapock rivers. Uncontrolled and mobile populations of undocumented gold miners could reintroduce malaria in the malaria-free coastal area of French Guiana, where competent vectors are abundant. The risk of an introduction of *P. vivax* malaria is probably higher, as *P. vivax* gametocytes are produced within the first cycles of infection [59]. This situation is challenging for malaria control and elimination in French Guiana, but also in all neighbouring countries of the Guiana Shield. There is a need to join and coordinate the efforts of all countries in order to successfully control malaria in the region.

## Conclusions

Despite a decrease in the incidence of malaria in French Guiana, uncontrolled foci of malaria transmission are present in the deep forest. Gold mining is associated with malaria transmission in all the countries of the Guiana Shield. Collaboration with neighbouring countries is necessary to take into account mobile populations such as gold miners. Military personnel are especially exposed during missions at illegal gold mining sites. Malaria control strategies in the French armed forces must be adapted to *P. vivax* malaria and sylvatic *Anopheles* mosquitoes. To that end, the French military health service and Institut Pasteur de la Guyane initiated a large research programme in 2012 to evaluate human, mosquito and parasite aspects of *P. vivax* malaria transmission at illegal gold mining sites in French Guiana.

## Abbreviations

RCP: river checkpoints (RCP)
FOB: forward operational bases on main rivers
FOP: forward operational post in deep forest
MM traps: Mosquito Magnet^®^ traps
CDC traps: Centers for Disease Control and Prevention light traps
PCR: polymerase chain reaction
ITS2: mosquito internal transcribed spacer 2
CSP: circumsporozoite protein
